# Environmentally compatible bioconjugated gold nanoparticles as efficient contrast agents for inflammation-induced cancer imaging

**DOI:** 10.1186/s11671-019-2986-y

**Published:** 2019-05-17

**Authors:** Vinícius Barreto Garcia, Thaís Gomes de Carvalho, Luiz Henrique da Silva Gasparotto, Heloiza Fernanda Oliveira da Silva, Aurigena Antunes de Araújo, Gerlane Coelho Bernardo Guerra, Timo Schomann, Luis J. Cruz, Alan B. Chan, Raimundo Fernandes de Araújo Júnior

**Affiliations:** 1 Department of Morphology, Federal University of Rio Grande do Norte, Natal, RN 59072-970 Brazil; 2 Post-Graduation Programme in Structural and Functional Biology, Federal University of Rio Grande do Norte, Natal, RN 59072-970 Brazil; 3 Post-Graduation Programme in Health Science, Federal University of Rio Grande do Norte, Natal, RN 59072-970 Brazil; 4 Department of Biophysics and Pharmacology, Post-Graduation Programme in Public Health, Post-Graduation Programme in Pharmaceutical Science, Federal University of Rio Grande do Norte, Natal, RN 59072-970 Brazil; 5 Group of Biological Chemistry and Chemometrics, Institute of Chemistry, Federal University of Rio Grande do Norte, Natal, RN 59072-970 Brazil; 6 Translational Nanobiomaterials and Imaging, Department of Radiology, Leiden University Medical Center, 2333 ZA Leiden, the Netherlands; 7 Percuros B.V, 2333 CL Leiden, the Netherlands

**Keywords:** Golden nanoparticles, M2 macrophages, Immunofluorescence, Inflammatory processes, Cancer diagnostics, Ulcerative colitis

## Abstract

For many cancers, early detection is the key to improve survival and reduce the morbidity, which is associated with radical resections due to late diagnosis. Here, we describe the efficiency of primary antibody-conjugated gold nanoparticles (AuNPs) to specifically target chronic inflammatory processes, specially M2 macrophages, in tissue sections of ulcerative colitis (UC) and steatohepatitis in rats which may lead to colorectal cancer and liver carcinoma, respectively. In this study, we demonstrate that AuNPs synthesized by a simple, inexpensive, and environmentally compatible method can be easily conjugated with the antibodies anti-COX-2, anti-MIF, and Alexa Fluor® 488 (ALEXA) to perform immunofluorescence staining in inflamed tissues. Moreover, we showed that primary antibody-conjugated gold nanoparticles (AuNPs) can be used to target M2 macrophages by flow cytometry. We designed three immunofluorescence staining protocols of tissue section with AuNPs for 30 min and overnight incubation, as well as one flow cytometry protocol of M2 macrophage labeling with AuNPs for 30 min. Immunofluorescence and flow cytometry results suggest that conjugation was achieved by direct adsorption of antibodies on the AuNPs surface. When compared to the standard ALEXA protocol in immunofluorescence (IF) and flow cytometry (FC), our 30-min incubation protocol using AuNPs instead of ALEXA decreased from approximately 23 h to 5 h for IF and from 4 h to 1 h for FC, proving to be less laborious, which makes the method eligible for inflammation-induced cancer diagnostic.

## Introduction

In medical and biological research, the interest in gold nanoparticles (AuNPs) for optical microscopy studies and diagnostic procedures, especially confocal laser microscopy, is increasing. The use of antibody/AuNP conjugates allows real-time detection of gold uptake into living cells (e.g., cancer cells) at the level of single particles, enabling the estimation of intracellular amounts of NPs [1–3].

The physicochemical properties of AuNPs allow them to be used in many medical studies, such as genomics, biosensitivity, immunoassay, clinical chemistry, detection, and photothermolysis of microorganisms and cancer cells [1]. Faulk and Taylor [4] described the first method of antibody conjugation with colloidal gold for direct electron microscopical visualization of the surface antigens of salmonellae. Since then, many studies were developed aiming at the application of nanoparticles conjugated with biomacromolecules, such as antibodies, lectins, and enzymes, in different fields, e.g., biochemistry, microbiology, immunology, and morphology [1, 4]. In cancer studies, highly specific and sensitive AuNP-based contrast agents are used for both X-ray and optical imaging modalities, since AuNPs are able to enhance fluorescence intensity of conjugated molecules [5, 6].

In 2014, we demonstrated that AuNPs can be applied to the indirect immunofluorescence (IF) staining method in cell cultures [6]. In the present study, we describe three new methods for immunofluorescence (IF) staining using AuNPs. Here, we use tissue sections and compare the fluorescence intensity from each of these methods to the standard staining protocol with Alexa Fluor® 488 (ALEXA) antibody (A1) with the goal of optimizing a fluorescent technique for clinical application [7–9].

Fluorescence imaging (FI) for cancer cell targeting utilizes a variety of optical imaging technologies in order to improve the detection of early neoplasia based on molecular signatures specific for cancer [10]. Since 2013, there has been a rapid increase in the number of clinical trials using FI. Screening is generally considered for high-risk patients, which is based on a combination of lifestyle factors, genetics, or a personal history of disease, while surveillance is reserved for patients with a diagnosis of dysplasia/chronic inflammation or those with suspected malignancy. FI may aid in identifying malignant lesions with improved specificity and sensitivity compared to currently available techniques. Furthermore, FI-based screening may provide a less invasive, more cost-effective way to detect cancerous or pre-cancerous lesions. Specifically, the ability of FI to detect lesions earlier than conventional screening methods will not only result in improved treatment outcomes but also in reduced treatment costs as it will prevent the need for multimodality care, which is required for those diagnosed at later diagnosis [11]. In this study, we demonstrate that antibody/AuNPs conjugates can successfully be applied for imaging of chronic inflammation by means of fluorescence microscopy.

## Methods/Experimental

### Aim of the Study

The aim of this study is to demonstrate how fluorescence properties of gold nanoparticles can be used in IF and flow cytometry techniques. In addition, we compared the protocols tested with AuNPs to the standard protocol using ALEXA and proved that it is possible to use the AuNPs in a protocol, which is faster, but as reliable as the standard protocol.

### Chemicals and Reagents

Gold trichloride (30 wt% in HCl), polyvinylpyrrolidone (PVP, MW = 10.000), sodium hydroxide, dialysis tubing cellulose membrane, and glycerol were products of Sigma-Aldrich Chemical Co (Saint Louis, USA). Sulfuric acid and hydrogen peroxide were purchased from Vetec (Rio de Janeiro, Brazil). Phosphate-buffered saline (PBS) solution and bovine serum albumin (BSA, 5%) were purchased from Life Technologies Corporation© (California, USA). Anti-COX-2 (cyclooxygenase-2**)** and anti-MIF (macrophage migration inhibitory factor) primary antibodies were acquired from Santa Cruz Biotechnology (São Paulo, Brazil), whereas Goat Anti-Rat IgG H&L Alexa Fluor® 488 secondary antibody was purchased from ABCAM® (Cambridge, UK). Fluoroshield Mounting Medium with DAPI (20 ml) from ABCAM® (Cambridge, UK) was used for counterstaining of DNA.

### Production and Characterization of AuNPs and Fluorescence Spectroscopy

Spherical AuNPs (7.1-nm) were produced and characterized according to a study by Gasparotto et al. [12]. Briefly, all glassware was kept in KMnO_4_ + NaOH solution overnight, rinsed with deionized water, kept in H_2_O_2_ + H_2_SO_4_ solution (1:1 *v*/*v*) for 10 min, again rinsed with deionized water, and dried prior to use. PVP (0.20 g) and gold chloride (6.80 mg) were dissolved in 10 ml of water. In a separate beaker, glycerol (0.18 g) and NaOH (0.080 g) were dissolved in 10 ml of water. The glycerol-NaOH solution was then added to the AuCl_3_-PVP solution to yield the following final concentrations: 1.0 mmol/L^−1^ Au^3+^, 0.10 M NaOH, 0.10 M glycerol, and 10 g/L^−1^ PVP. The final mixture had a deep-red color due to the formation of AuNPs. The colloidal ultraviolet-visible absorption spectra of the AuNPs were acquired with an Evolution 60S UV–visible spectrophotometer (Thermo Scientific, MA, USA). Fluorescence spectroscopy was carried out with a RF-5301 PC spectrofluorophotometer (Shimadzu, Kyoto, Japan).

### Experimental Design

All three protocols were tested on control groups of two chronic inflammatory models, acetic acid-induced ulcerative colitis (UC) [7], and alcohol-induced steatohepatitis [8, 9] in rats.

Acetic acid-induced UC was performed on female Wistar rats (220 ± 20 g of BW), obtained from the Biotechnology Center/Universidade Federal da Paraiba. The animals were divided into two groups (*n* = 5 per group): acetic acid control and non-colitic rats. The animals were fasted overnight and anesthetized with ketamine (70 mg/kg, 10%) and xylazine (10 mg/kg, 2%). UC was induced according to methods originally described by MacPherson and Pfeiffer [13] and modified by Millar et al. [14]. A catheter was carefully inserted into the colon. Then, 10% acetic acid (0.5 ml) in 0.9% saline was instilled into the lumen of the colon. The non-colitic group received 0.5 ml of 0.9% saline intracolonically. The rats were maintained in a supine Trendelenburg position for 30 s to prevent leakage of the intracolonic instillation.

The animals were fasted overnight and euthanized with an overdose of thiopental, 48 h after induction. Next, the colon was removed aseptically, rinsed with PBS, and placed on an ice-cold plate. The colon was cleaned of fat and mesentery. Then, each specimen was weighed and its length measured under a constant load (2 g). Afterward, the colon was opened longitudinal and scored for macroscopically visible damage on a scale of 0 to 10 according to the criteria described by Bell et al. [15].

Alcohol steatohepatitis was induced in male Wistar rats (290 ± 10 g of BW) obtained from the Department of Biophysical and Pharmacology – Federal University of Rio Grande do Norte (UFRN). The animals were divided into two groups (*n* = 5 per group): alcoholic and non-alcoholic rats. Ethanol solution (7 g/kg body weight of 30% *v*/*v*) was used as a chronic dose for the alcoholic group. Animals in the non-alcoholic group orally received an equivalent volume of saline solution (0.9% NaCl) by gavage. Gavage procedures were performed once a day in both groups for 28 days.

On day 29, euthanasia was performed by intraperitoneal injection of 7.5 ml/kg ketamine (50 mg/ml) and 2.5 ml/kg Xylazine (20 mg/ml). Before euthanasia, all animal groups were fasted for 12 h. Once unconscious, the animals underwent cardiac puncture followed by removal of the liver. Liver fragments were immersed in 10% buffered formaldehyde for histopathological analysis.

Animals from both acetic acid-induced UC and alcohol-induced steatohepatitis were housed under standard conditions (12-h light/dark cycle, 22 ± 0.1 °C, and 50–55% humidity) with *ad libitum* access to food and water. Animals were treated according to the ethical principles for animal experimentation.

### Histology

Five paraffin blocks from the positive controls (acetic acid control and alcoholic control) of each inflammation model were used for indirect IF staining. The primary antibodies anti-COX-2 and anti-MIF were found to be good inflammation markers in acetic acid-induced UC and alcohol-induced steatohepatitis, respectively, by providing a reliable staining in terms of fluorescence intensity that can be used to compare different protocols.

The tissues were fixed in 10% buffered formaldehyde, dehydrated with ethyl alcohol (70%, 80%, 90%, 95%, and P.A.), clarified in Xylol and impregnated in paraffin following the standard protocol [8]. Prior to the preparation of the sections for indirect IF staining, the slides were kept in an oven at 60 °C for 24 h.

### Immunofluorescence and Protocols Designs

**Fig. 1:**
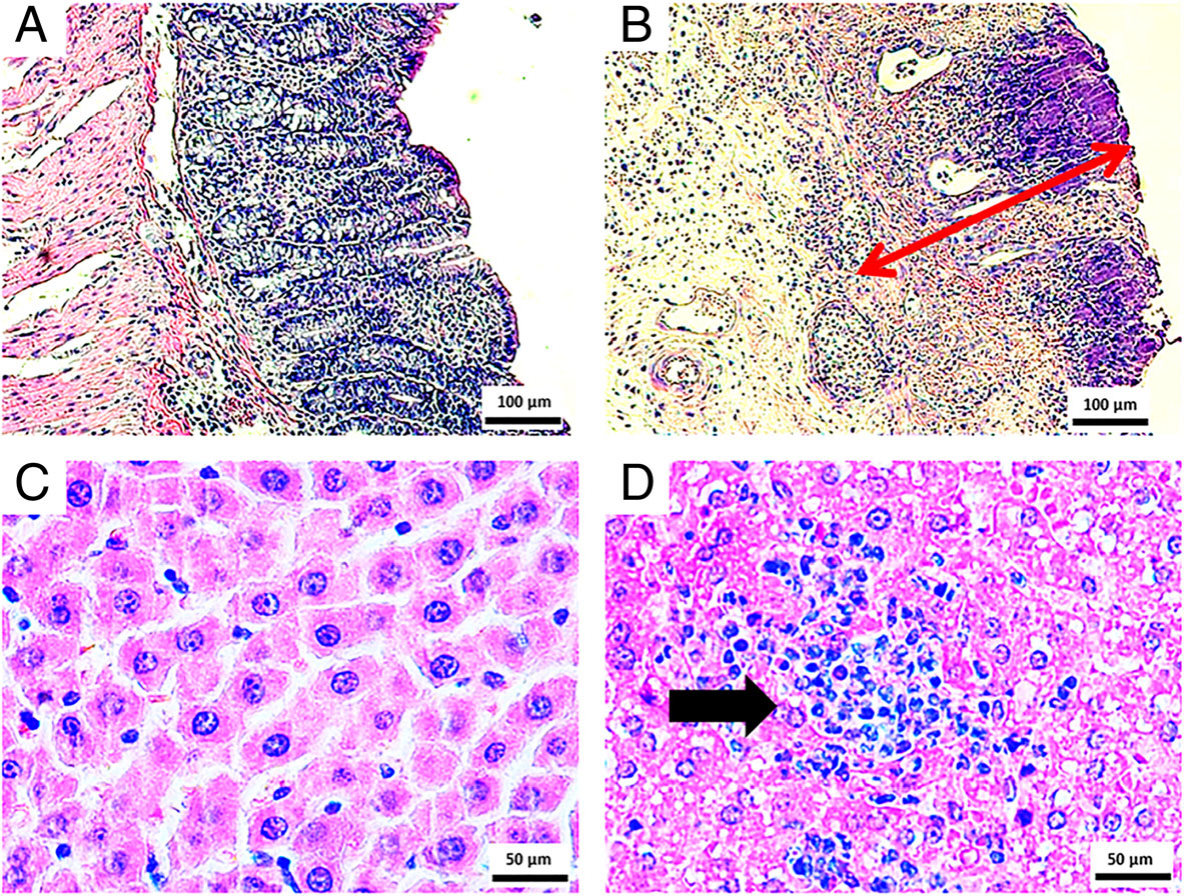
Images of hematoxylin and eosin staining of acetic acid-induced UC and alcohol-induced steatohepatitis control groups. **a** Negative control of UC Model (× 100; scale bar = 100 μm). **b** Acetic acid-induced UC. The red arrow indicates leukocyte infiltration of the mucosa area (× 100; scale bar = 100 μm). **c** Negative control of steatohepatitis (× 200; scale bar = 50 μm). **d** Alcohol-induced steatohepatitis. The black arrow indicates leukocyte infiltration (× 200; scale bar = 50 μm)

Three tissue sections (4 μm) from each animal were deparaffinized in xylene and washed in a series of decreasing concentrations of ethanol, from 99% ethanol to 50% ethanol. Finally, tissue sections were washed twice in dH_2_O and one wash with PBS. Antigen retrieval was performed by placing the sections in a 10 mM sodium citrate with 0.05% Tween 20 at 95 °C for 30 min and subsequently at room temperature (RT) for 20 min. Background noise/signal was reduced by incubating the sections in 0.1% Sudan black in 70% alcohol at RT for 20 min, followed by three washes in 0.02% PBS-Tween 20. The samples were permeabilized in 0.2% Triton-X-100 in PBS (three washes, 5 min each), washed in PBS, and then blocked with PBS, 5% BSA, dH_2_O, and Triton-X-100. Slides were incubated with block solution in a humidity chamber for 2 h. As shown in Table 1 and Fig. 1, the incubation of the anti-COX-2 and anti-MIF primary antibodies was performed following three different protocols (NP1, NP2, and NP3). These protocols were compared to two ALEXA-based protocols that differ in the time of incubation of the primary antibody—the ALEXA standard protocol (overnight incubation) adopted by our research group as standard protocol for most procedures (A1) and ALEXA modified protocol (A2; 30 min incubation). NP1 is a nanoparticle-dependent immunofluorescence staining method with 30 min for primary incubation. Primary antibodies were diluted in 1% BSA at ratios of 1:500 (anti-COX-2) and 1:400 (anti-MIF), and each sample was incubated in a humidity chamber at RT (20 °C) for 30 min. Next, 100–150 μl of AuNPs were added to the samples, which were left incubated at RT (20 °C) for another 30 min. In the second nanoparticle-dependent protocol (NP2), primary antibodies were diluted in 1% BSA and applied directly to the slides, which were left in the refrigerator overnight. Therefore, A2 and NP1 are both 30-min incubation protocols, but A2 is performed with a secondary fluorescent antibody (ALEXA), whereas NP1 has AuNPs as fluorophores. This also applies for the overnight-incubation protocols A1 and NP2. After the respective incubation times, the slides of each protocol were washed three times with PBS to remove the excess primary antibody. Then, 100–150 μl of nanoparticles were added to each slide in NP2, whereas ALEXA (1:400) was added in A1. After 1 h of incubation with AuNPs (NP2) and ALEXA (A1), all slides were washed three times with PBS.

**Table 1:** Comparison of immunofluorescence protocols based on ALEXA and AuNPs

	Deparaffinization, rehydration and PBS washes.	Antigen Retrieval - block solution	Primary antibodies incubation	ALEXA/ AuNPs incubation	PBS washes and slides assembling with DAPI
Immunofluorescence Protocols					
A1	1h	3,8h (230 min)	18h	ALEXA (1h)	20 min
A2	1h	3,8h (230 min)	30 min	ALEXA (30 min)	20 min
NP1	1h	3,8h (230 min)	30 min	AuNPs (30 min)	20 min
NP2	1h	3,8h (230 min)	18h	AuNPs (1h)	20 min
NP3	1h	3,8h (230 min)	18h	ALEXA + AuNPs (1h)	20 min

The third protocol (NP3) was design to explore the amplifying properties of AuNPs in fluorescent systems. In NP3, AuNPs were applied during dilution of ALEXA in 1% BSA. Three dilutions of secondary antibody (1:200, 1:400, and 1:800) in BSA with AuNPs were tested in order to compare the fluorescence intensities of each dilution to A1. The dilutions were prepared in such a way that the volume of AuNPs was proportional to the volume of BSA, e.g., for a 1:200 dilution, 2 μl of ALEXA was diluted in 199 μl of AuNPs + 199 μl of BSA. The incubation time for the primary antibodies in A1 and NP3 was approximately 18 h (overnight), whereas the incubation time for ALEXA + AuNPs suspension was 1 h.

Finally, samples were mounted using Fluoroshield Mounting Medium with DAPI. The slides were stored in a light-protected box and kept at 4 °C until microscopical analysis. Fluorescent images were obtained with a Zeiss Observer z.1 upright microscope for fluorescence and bright field imaging (× 20 and × 10 objectives, Carl Zeiss, Jena, Germany) with AxioCam MRc. The arithmetic mean parameter of the Zeiss ZEN lite blue edition software (Carl Zeiss) was utilized to quantitatively evaluate the intensity of fluorescence pixel by pixel for each picture of the ALEXA channel. At least three samples of each animal (five animals per group) were analyzed and five pictures were taken from each fragment with the × 10 objective.

### Induction of M2-polarized TAMs In Vitro

For evaluation of primary antibody-conjugated gold nanoparticles (AuNPs) in M2 macrophages, RAW 264.7 cells (5 × 10^5^ cells/well in a 12-well plate) were cultured in complete medium with 10% fetal bovine serum supplemented with 20 ng/ml IL-4 for 24 h. After treatment with IL-4, cells were washed three times with serum-free DMEM, followed by culture in the same medium for 48 h. Cells were analyzed using a Telaval 31 light microscope (ZEISS, Oberkochen, Germany).

### Flow Cytometry

After incubation with IL-4 for 48 h, RAW 264.7 cells and M2 macrophages (1.5 × 10^4^ cells/well in a 12-well plate) were collected with a scraper and blocked with 0.5 g BSA in 100 ml PBS (PBA) for 45 min, followed by incubation with PerCP-conjugated anti-mouse CD163 (1:1000) and FITC-conjugated anti-mouse CD86 (1:1000) at 4 °C for 60 min. Besides, M2 macrophages were labeled with COX-2 and MIF primary antibodies and so conjugated to AuNPs. M2-polarized TAMs were collected and fixed with 2% paraformaldehyde in PBS for 10 min, followed by incubation with the anti-COX-2 and anti-MIF primary (1:100) for 60 min at 4 °C and; afterward, the cells were labeled with goat anti-rabbit Alexa® Fluor 488-conjugated secondary antibody (Thermo Fisher Scientific) in incubation solution (1:100) at room temperature for 60 min as described in the Alexa standard protocol [16]. Following the protocol (N1) where AuNPs replace the goat anti-rabbit Alexa® Fluor 488-conjugated secondary antibody, M2 macrophages were harvested, washed with PBS, and incubated with the anti-COX-2 and anti-MIF primary (1:100) in incubation solution at room temperature for 30 min. Then, the cells were incubated with AuNPs (1:5) at 4 °C for 30 min (AuNPs excitation at 520 nm). Following the final washing step, labeled cells were analyzed in a BD FACSCanto II (BD Biosciences, the Netherlands) and FlowJo software (version 10.1; Tree Star Inc., UK). All flow cytometry analyses were performed with samples in triplicate and repeated three times. Comparison between standard ALEXA and AuNPs protocols are shown in Table 2.

**Table 2:** Comparison of flow cytometry protocols based on ALEXA and AuNPs

	Overral time	2% Formaldehyde fixation (10 min, 37°C)	Primary antibody incubation	Incubation with fluorochromeconjugated substances	Washes	Resuspension and analysis
Flow Cytometry Protocols						
Standard	4h	Yes	1h	ALEXA 488 diluted in PBA - 1 h	2x	Yes
AuNPs	1h	No	30 min	AuNPs - 30 min	None	Yes

### Statistical Analysis

Analysis of variance (ANOVA) and Bonferroni’s *post hoc* test were performed for immunofluorescence analysis. For flow cytometry analysis, the significant differences between the groups were calculated using ANOVA and Dunn’s test, as indicated. A *p* < 0.05 was considered statistically significant for all analysis performed.

## Results

### Microscopy Analysis

The section in Fig. 1a shows a tissue section stained with hematoxylin and eosin (negative control). The chronic inflammatory process induced by acetic acid is depicted in Fig. 1b, which reveals loss of tissue architecture with consequent destruction of the epithelium, a reduction in goblet cells, the presence of hemorrhages and leukocytes near the injured sites. In figure fatty droplet accumulation and inflammatory infiltrate (neutrophils and lymphocytes) were seen in the livers from rats subjected to chronic alcohol exposure.

**Fig. 2:**
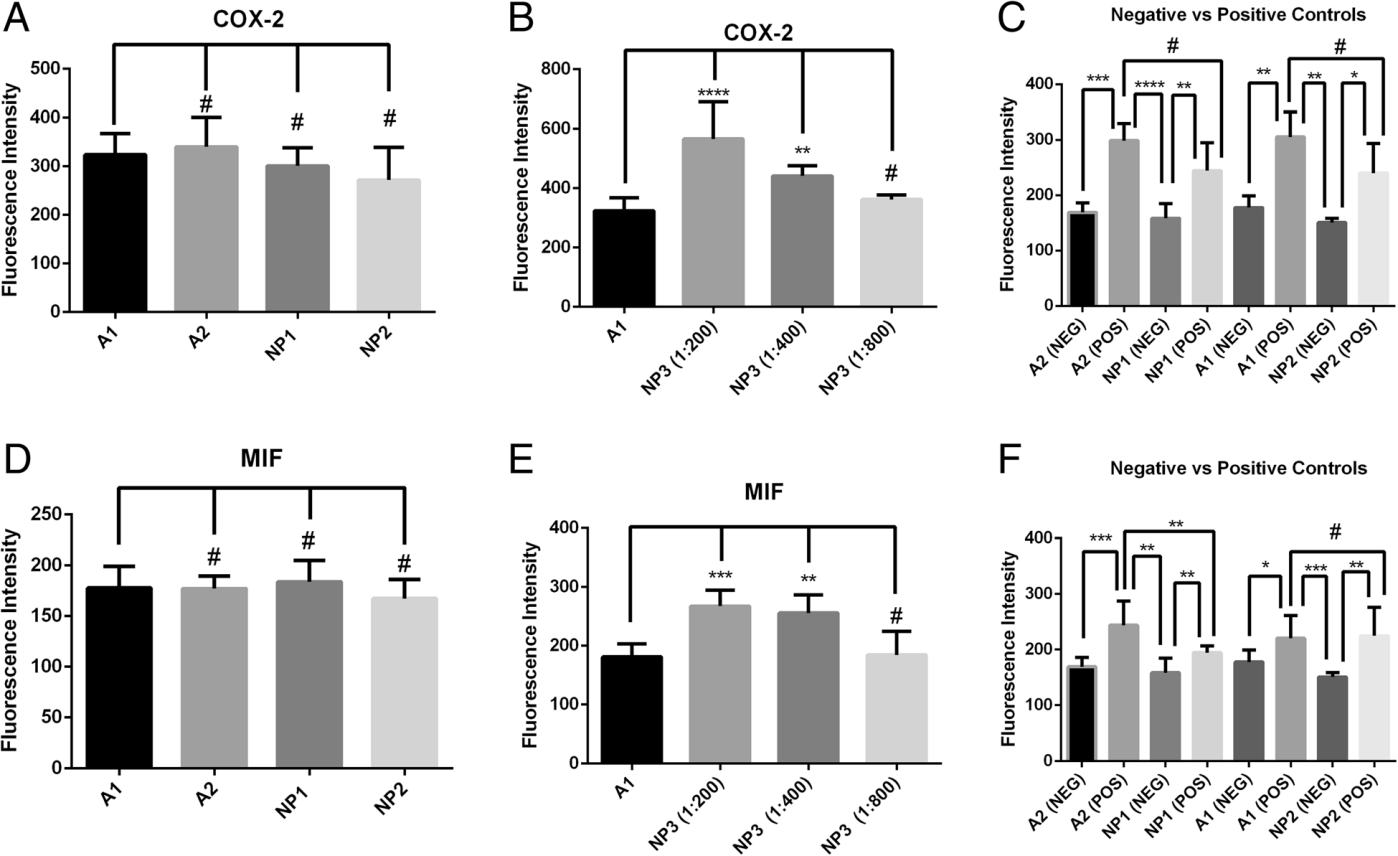
Comparison of fluorescence intensity among groups. **a** Fluorescence intensity of AuNPs in A2, NP1, and NP2 in comparison to A1-only acetic acid-induced UC (anti-COX-2 antibody). **b** All dilutions of NP3 (1:200, 1:400, and 1:800) in comparison to the fluorescence intensity of A1 (1:400)-only acetic acid-induced UC (anti-COX-2 antibody). **c** Comparison of the fluorescence intensity among acetic acid-induced animals and negative controls after short-time (A2 and NP1) and overnight incubation (A1 and NP2). **d** Fluorescence intensity of AuNPs in A2, NP1, and NP2 in comparison to A1-only alcohol-induced steatohepatitis (anti-MIF antibody). **e** All dilutions of NP3 (1:200, 1:400, and 1:800) in comparison to the fluorescence intensity of A1 (1:400)-only alcohol-induced of steatohepatitis (anti-MIF antibody). **f** Comparison of the fluorescence intensity among alcohol-induced steatohepatitis animals and negative controls after 30-min incubation (A2 and NP1) and overnight incubation (A1 and NP2). Statistics: ANOVA and Bonferroni’s post hoc test, ^#^*p* ≥ 0.05, **p < 0.01,***p < 0.001, and ****p < 0.0001

In terms of fluorescence intensity, AuNPs looked similar to ALEXA, which is a conventional fluorophore for IF staining. In both liver and colon samples, the differences in the fluorescence intensity of NP1 and NP2 were minimal or non-existent when compared to A1. In Fig. 2, multiple comparisons among the IF protocols tested using anti-COX-2 and anti-MIF primary antibodies are shown. Figures 2a and d demonstrate that the fluorescence intensities of A2, NP1, and NP2 are not significantly different in comparison to A1 protocol (^#^*p* > 0.05), suggesting that 30-min incubation protocols (either using ALEXA or AuNPs) may be applicable in a diagnostic context.

In addition, the NP3 protocol (Fig. 2b, e) demonstrated that it is possible to combine the AuNPs to ALEXA so that the detected fluorescence intensity was increased (***p < 0.001), allowing even double of the dilution of ALEXA without the fluorescence decreasing relative to the 1:400 dilution originally tested on A1 (^#^*p* > 0.05).

**Fig. 3:**
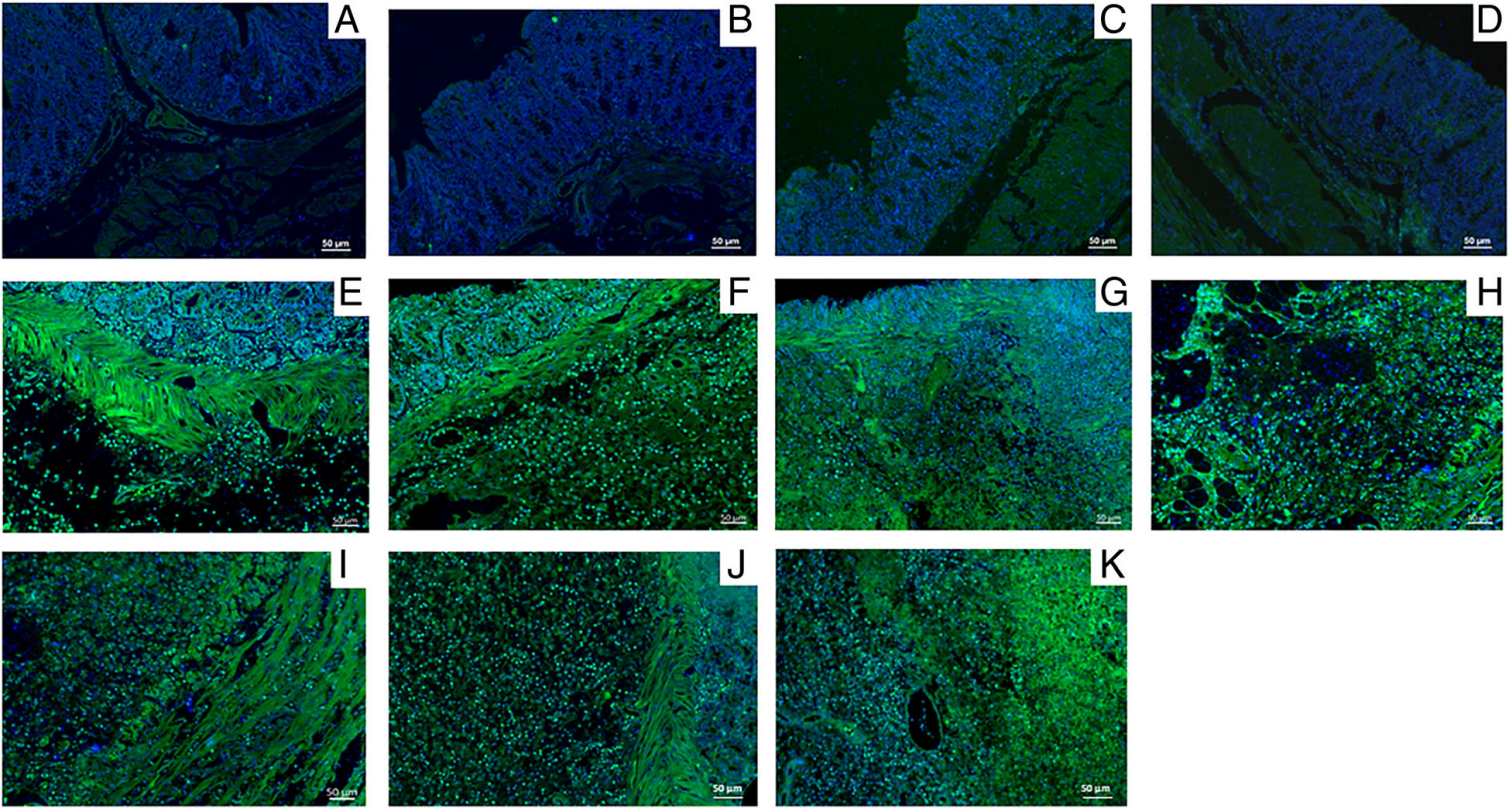
Anti-COX-2 IF images of colon samples from the saline (negative control) and the acetic acid-induced UC groups stained with green fluorescent compounds (ALEXA, AuNPs, or both) and DAPI (blue) for nuclei. **a** Negative control stained with ALEXA standard protocol (A1) with anti-COX-2 and ALEXA (green). **b** Negative control stained with modified ALEXA protocol (A2) with anti-COX-2 and ALEXA. **c** Negative control stained with nanoparticle protocol 1 (NP1) with anti-COX-2 and AuNPs (green) instead of ALEXA. **d** Negative control stained with nanoparticle protocol 2 (NP2) with anti-COX-2 and AuNPs instead of ALEXA. **e** Positive control stained with A1. **f** Positive control stained with A2. **g** Positive control stained with NP1. **h** Positive control stained with NP2. **i** Positive control stained with nanoparticle protocol (NP3) with anti-COX-2 + AuNPs with 1:200 ALEXA. **j** Positive control stained with NP3 (with 1:400 dilution of ALEXA). **k** UC positive control stained with NP3 (with 1:800 dilution of ALEXA). Magnifications, × 200. Scale bar = 50 μm

**Fig. 4:**
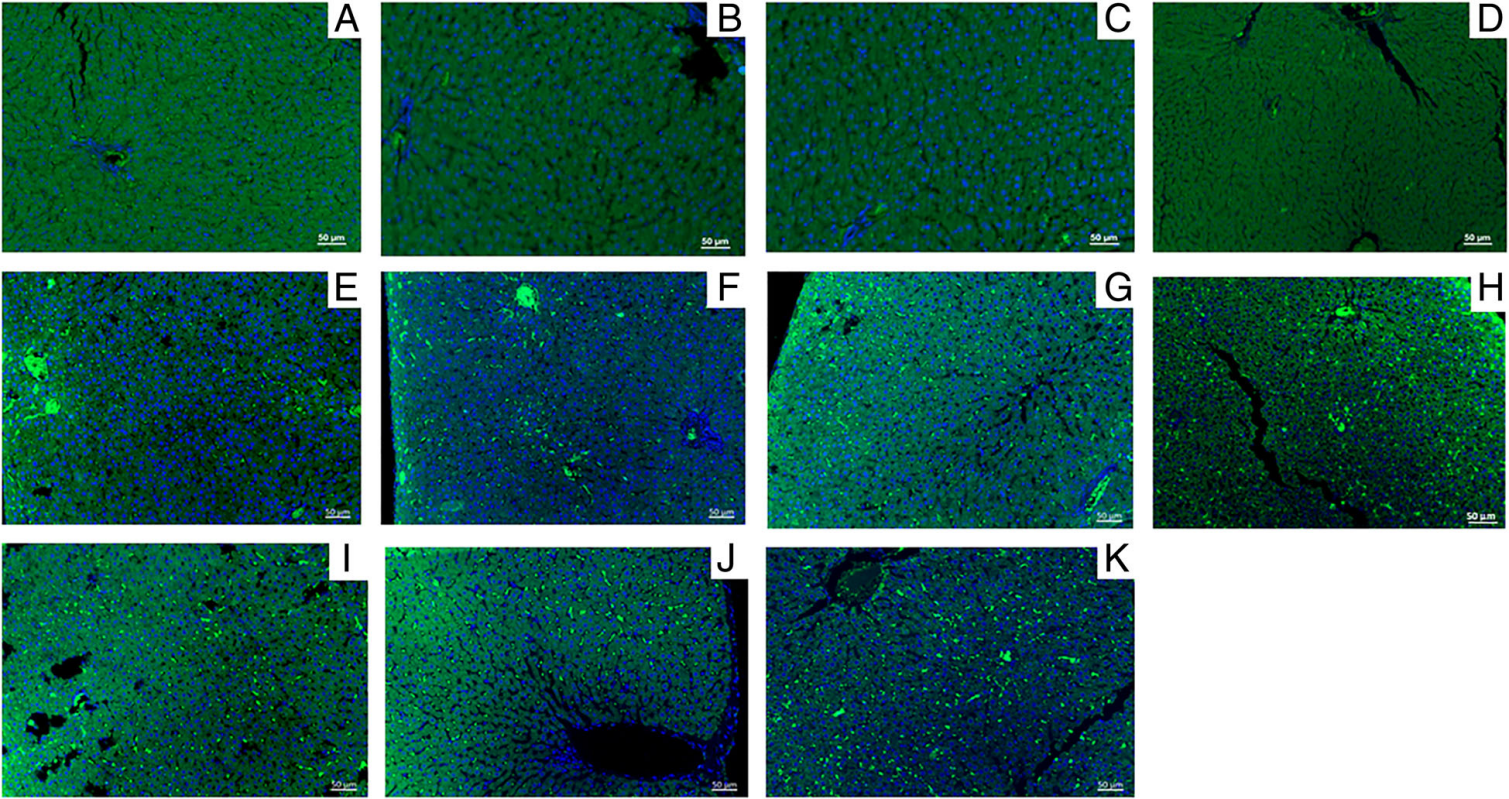
Anti-MIF IF images of liver samples from the saline (negative control) and alcohol-induced steatohepatitis groups stained with green fluorescent compounds (ALEXA, AuNPs, or both) and DAPI (blue) for nuclei. **a** Negative control stained with ALEXA standard protocol (A1) with anti-MIF and ALEXA (green). **b** Negative control stained with ALEXA modified protocol (A2) with anti-MIF and ALEXA. **c** Negative control stained with nanoparticle protocol 1 (NP1) with anti-MIF and AuNPs (green) in place of ALEXA. **d** Negative control stained with nanoparticle protocol 2 (NP2) with anti-MIF and AuNPs in place of ALEXA. **e** Positive control stained with A1. **f** Positive control stained with A2. **g** Positive control stained with NP1. **h** Positive control stained with NP2. **i** Positive control stained with nanoparticle protocol 3 (NP3) with anti-MIF + AuNPs with 1:200 ALEXA. **j** Positive control stained with NP3 with a 1:400 dilution of ALEXA. **k** Positive control stained with NP3 with a 1:800 dilution of ALEXA. Magnifications, × 200. Scale bar = 50 μm

Finally, we compared the diseased groups to the healthy groups (Fig. 2f) to demonstrate that all protocols tested, either with ALEXA or with AuNPs, are effective in detecting the onset of inflammatory processes characterized by antigens COX-2 and MIF. Figures 3 and 4 support the data shown graphically in Fig. 2. Both ALEXA and AuNPs are represented in green staining, whose distribution corresponds to sites of increased leukocyte infiltration and tissue injury.

In terms of clinical application, the NP1 protocol proved to be the most promising, since, when compared to the standard A1 protocol, it allows a saving of 18 h.

### Flow Cytometry: Primary Antibody-Conjugated Gold Nanoparticle (AuNP)-Labelled M2 Macrophages

**Fig. 5:**
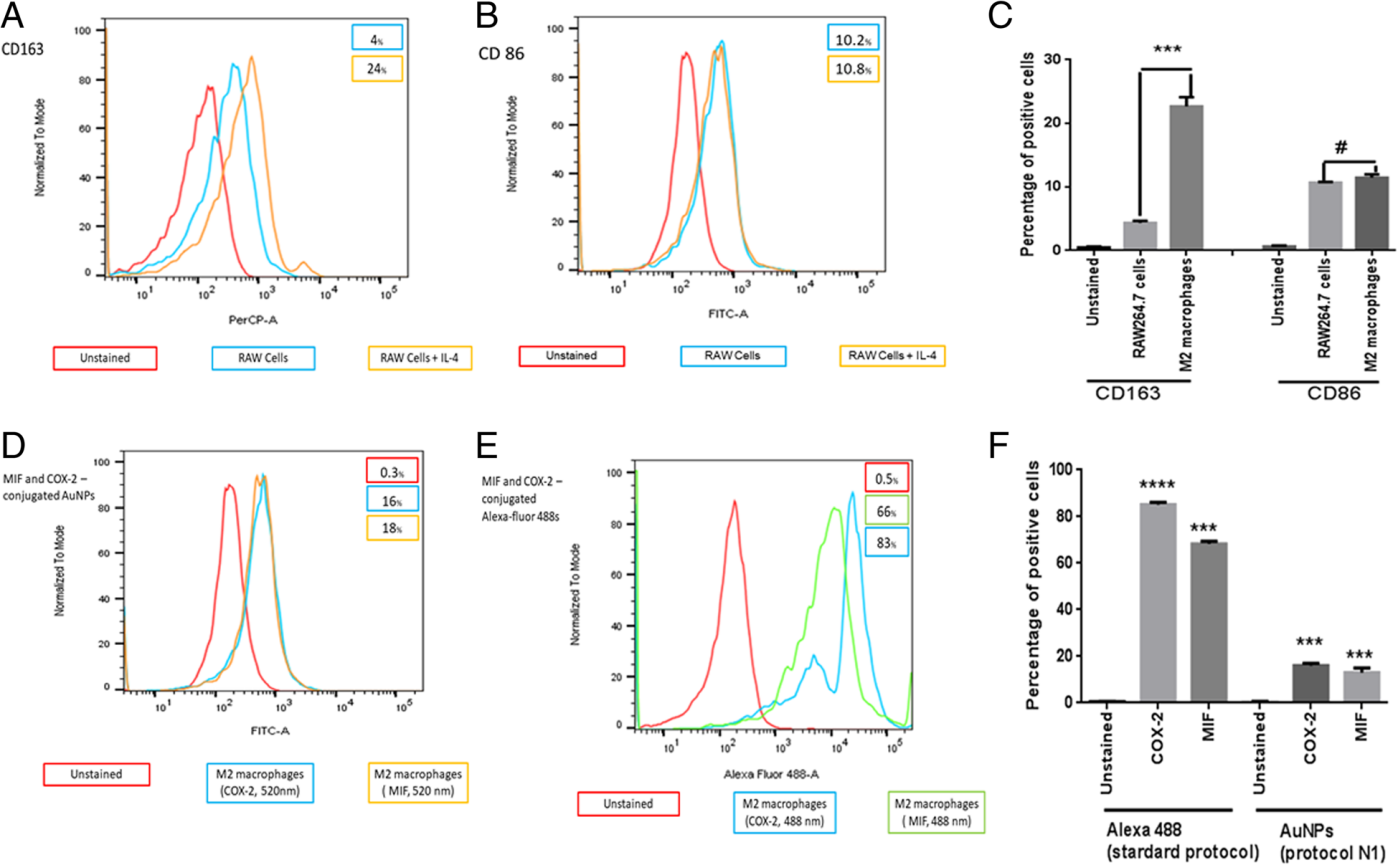
COX-2 and MIF antibody-conjugated gold nanoparticles (AuNPs) have high affinity on the surface of M2 macrophages. **a**–**c** RAW 264.7 cells were stimulated with IL-4 (20 ng/ml) for 24 h, followed by flow cytometry analysis to quantify the amount of CD163, an M2 macrophage marker, and CD86, an M1 marker. **d**, **f** Either COX-2 and MIF antibody-conjugated gold nanoparticles or **e**, **f** ALEXA 488 showed higher fluorescence intensity in M2 macrophages when compared to unstained samples. Data are expressed as mean ± SD, ^#^*p* > 0.05, ***p < 0.001, *p* < 0.0001. Representative flow data shown are from experiments independently performed at least three times

To further characterize M2 macrophages, the expression of M1 (CD86)- and M2 (CD163)-related makers in IL-4-stimulated RAW 264.7 cells were analyzed in flow cytometry. Flow cytometry results showed that the M2-related markers such as CD163 receptor were significantly higher than the native cell (Fig. 5a, c, *p* < 0.001) while CD86 receptor expression did not show any difference between M2 macrophages and naive cells (Fig. 5b, c, *p* > 0.05). The results suggested that IL-4 (20 ng/ml) successfully induced the alteration of classical macrophages to M2 macrophages. After this step, M2 macrophages were incubated with COX-2 and MIF primary antibodies and then labeled with AuNPs to compare the efficiency of primary antibody-conjugated gold nanoparticles (AuNPs) to the standard protocol (ALEXA). The results showed that either COX-2 and MIF antibody-conjugated gold nanoparticles (Fig. 5d, f, *p* < 0.001) or ALEXA (Fig. 5e, f, *p* < 0.001) showed higher fluorescence intensity in M2 macrophages when compared to unstained samples.

### Fluorescence Spectroscopy

**Fig. 6:**
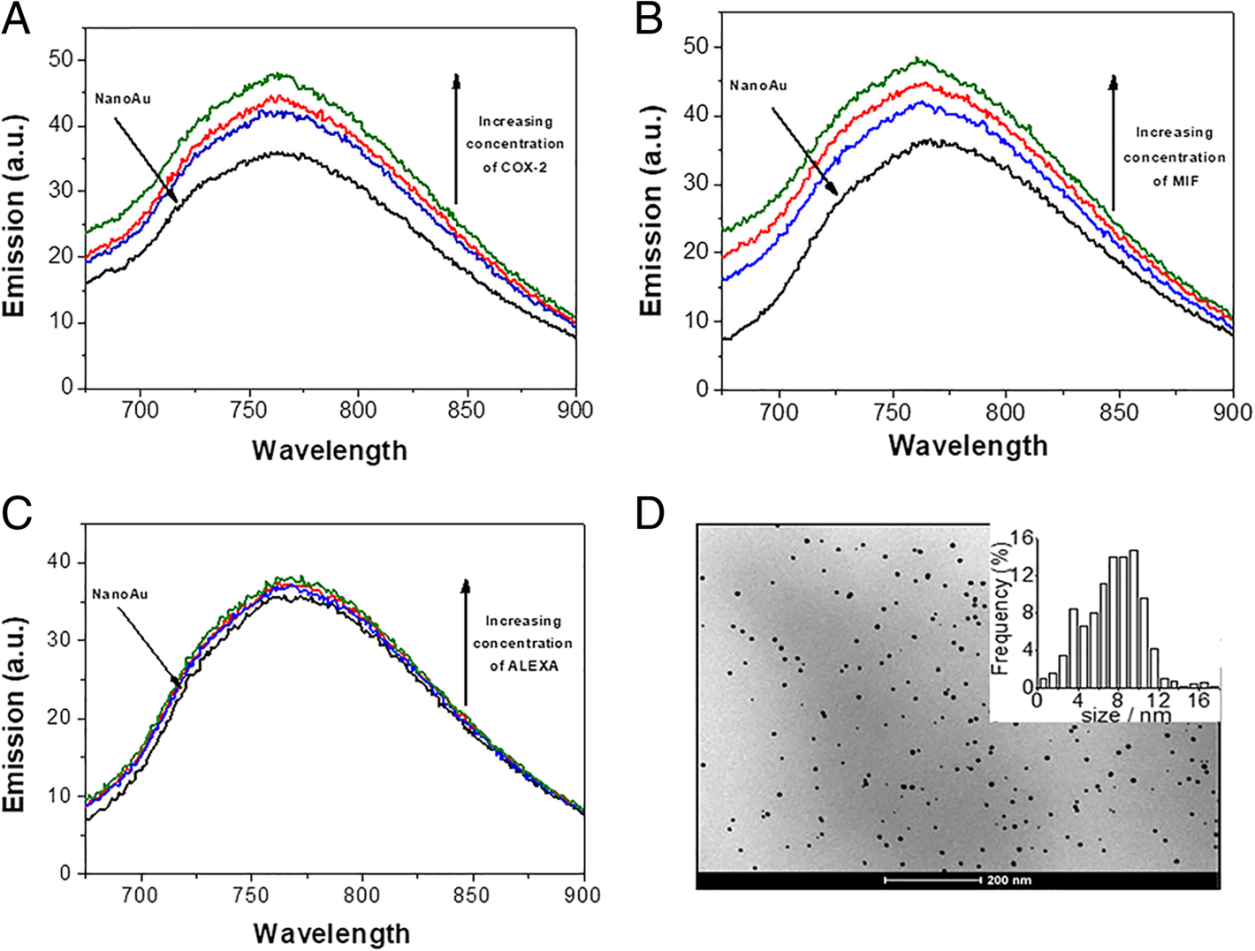
Fluorescence emission spectra of AuNPs excited at 320 nm in the presence of anti-COX-2 (**a**), anti-MIF (**b**), and ALEXA (**c**). The TEM image of the spherical nanoparticles used in this study, showing its size and distribution (**d**). Excitation and emission slits were 10 nm. The concentration of AuNPs was held constant at 29.6 ng/ml^−1^. The concentrations of antibody are 100 ng/ml^−1^ (blue curve), 150 ng/ml^−1^ (red curve), and 200 ng/ml^−1^ (green curve)

In order to measure the fluorescence emission spectra for purified AuNPs in the absence and presence of anti-COX-2 (Fig. 6a), anti-MIF (Fig. 6b), and ALEXA (Fig. 6c), the excitation wavelength was fixed at 320 nm and the emission was recorded in the range of 650 to 900 nm. An increase of the excitation wavelength did not change the emission range of the particles, thus excluding the possibility of a scattering process. Fluorescence emissions were slightly increased with anti-COX-2 uptake (increased 11.93 absorbance units (a.u.) for the highest antibody concentration), anti-MIF (increased of 12.15 a.u. for the highest antibody concentration), and ALEXA (increased of 3.18 a.u. for the highest antibody concentration) in combination with AuNPs.

## Discussion

It has been shown that AuNPs have fluorescence properties, due to their chemical structure and size, and can act as amplifying molecules of fluorescent signals. More specific, AuNP fluorescence itself originates from aurophilic interactions on the surface of gold atoms [17, 18]. The data from Fig. 6 is similar to the findings of studies on silver nanoparticles, which owe the increased fluorescence emission to the competition between adsorbent species and the molecular oxygen dissolved in solution. Therefore, the increased fluorescence emission of gold nanoparticles could be attributed to the fact that BSA is adsorbed on their surface [19].

The fluorescence signal is a function of the surface plasmon resonance phenomenon (SPR). Hence, the increase in the amount of free electrons leads to a more intense SPR signal, since SPR is the collective oscillation of free electrons. The adsorbed BSA prevents the surface free electrons from binding to the oxygen molecules, leading to an increase of the fluorescence signal [20, 21]. Anti-COX-2, anti-MIF, and ALEXA may have been adsorbed on the AuNPs, thereby preventing O_2_ from reaching the surface of the nanoparticles. This is supported by Fig. 6, where the increase of the fluorescence signal occurred at very low concentrations of the different antibodies. It is worth to notice, however, that ALEXA required a higher concentration to cause O_2_ isolation of the AuNPs surface.

The chronic inflammation process is characterized by the infiltration of immune cells, mainly macrophages, at the sites of injury caused by acetic acid or ethanol in the respective model. Inflammation is also characterized by the leukocyte-dependent release of several cytokines and mediators in response to a pathogen or a stressful condition. Among these cytokines, COX-2 and MIF are known to be markers for several inflammation processes, since they are pro-inflammatory cytokines. The green staining observed in Fig. 3 and Fig. 4 is due to the release of COX-2 and MIF cytokines, respectively.

In chronic UC and liver steatohepatitis, macrophages are the main cells found which can be classified into two major types: M1 macrophages and M2 macrophages. The classically activated macrophages (M1 macrophages) are proinflammatory and play a pivotal role in host defense against infection which is associated with iNOS and IL-23 production and their cell surface-expressed CD86 or HLA-DR that attract killer cells like neutrophils and/or direct Th1 (cytotoxic) responses and stimulate further M1-type responses [22]. Meanwhile, the alternatively activated macrophages (M2 macrophages) are associated with the responses to anti-inflammatory reactions as well as tissue remodeling [23]. In the tumor context, it has been documented that M2-polarized macrophages promote pro-tumor functions by production of a large array of growth factors such as COX-2 and MIF for tumor cells, which are essential for tumor proliferation [24].

It is well established that UC is an important risk factor for colonic epithelial dysplasia and adenocarcinoma [25, 26]. According to Agoff et al. [25], increased malondialdehyde (MDA) levels and upregulation of Bcl-2 during UC are potential mechanisms to explain the relationship between COX-2 overexpression and neoplastic progression. In UC, the inflammation boosts COX-2 activity, leading to genetic damage through increased production of MDA. MDA is a by-product of COX-mediated prostaglandin synthesis and lipid peroxidation, and it is also constitutively produced by COX-1. Since COX-2 upregulates Bcl-2 expression, it leads to resistance to apoptosis in UC-associated neoplasia [27].

MIF is a multipotent cytokine in the innate immune responses that contributes to hepatic injury driven by alcohol-induced steatohepatitis [28]. When steatohepatitis has developed, the liver morphology rarely goes back to normal, even after cessation. In addition, there is a higher risk of the development of cirrhosis, which is the last stage of alcoholic liver disease (ALD) before hepatocellular carcinoma (HCC) [29].

Studies show MIF presence in the sera after hepatic resection or expression in the course of liver cancer progression [30]. MIF is involved in COX-2 and PGE2 upregulation and directly promotes tumorigenesis by inhibition of p53 accumulation, which is a classic tumor suppressor gene that can promote cell cycle arrest and apoptosis in response to DNA damage [31].

In a previous study [6], we have demonstrated that AuNPs can be easily conjugated with the antibodies anti-β-catenin and anti-E-cadherin to specifically target colorectal carcinoma cells, whose clinical value can be found in an early diagnosis of cancer through non-invasive methods in body fluids such as saliva and urine. In addition, we developed a new protocol to decrease the 27 h that are usually needed for the standard protocol to about 1 h needed for our improved protocol, which makes this method eligible for a clinical colorectal cancer diagnostic.

**Fig. 7:**
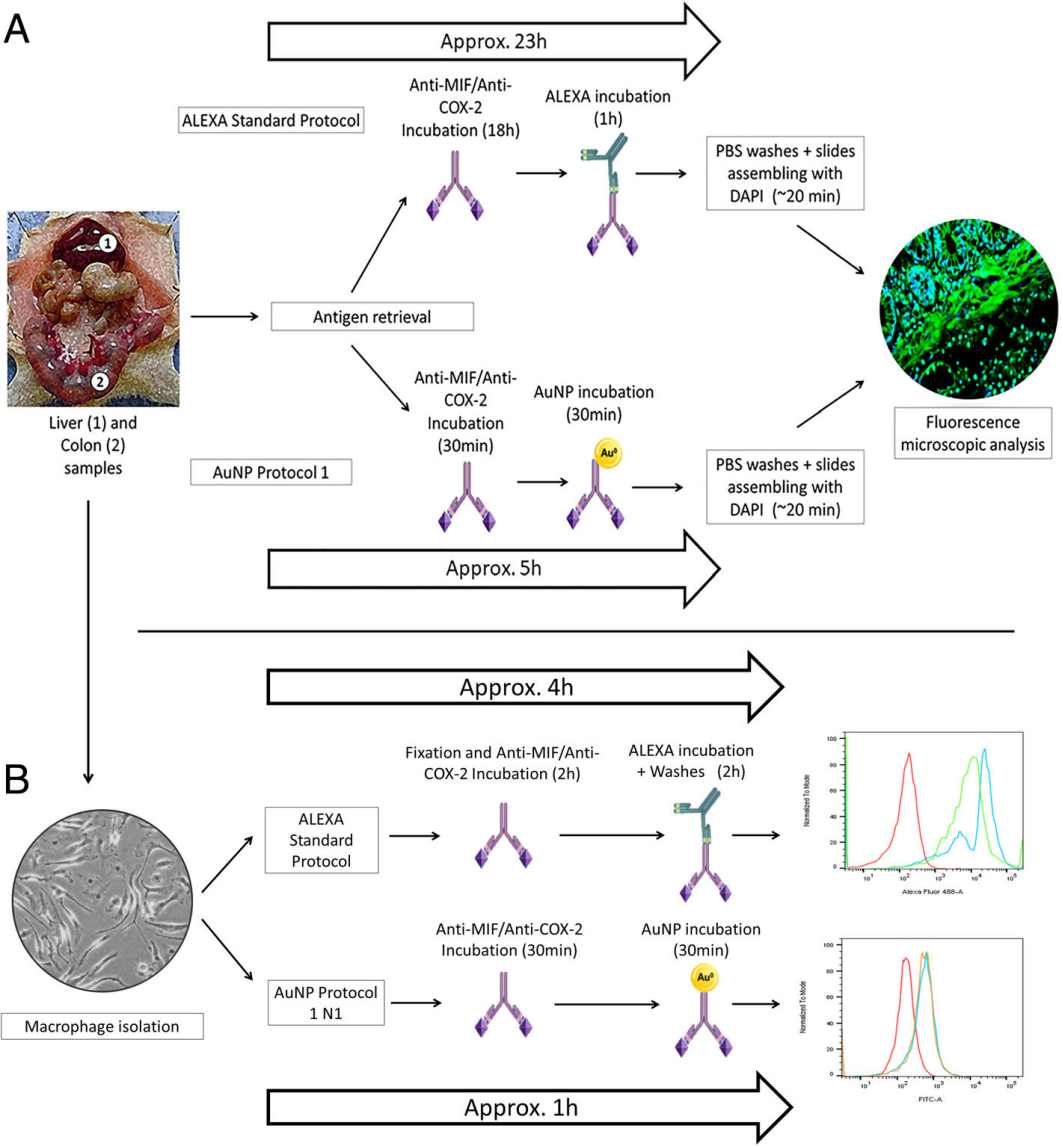
Comparative schemes illustrating the immunofluorescence (**a**) and flow cytometry protocols used in this study (**b**). In immunofluorescence, AuNPs may be applied in 30-min incubation protocols as fluorescence-enhancing agents, providing faster results with comparable fluorescence levels to the traditional ALEXA protocol, which makes NP1 a suitable protocol for cancer diagnose. Time was counted from the antigen retrieval to the end of the second incubation. In flow cytometry, although the ALEXA marking was higher than that of the AuNPs, the N1 protocol with AuNPs allowed a greater saving of reagents as well as reduced the time required for the technique from 4 to 1 h

In this study, we took advantage of the properties of AuNPs conjugated with primary antibodies and applied them for the indirect IF staining method of tissue sections. At this point, we cannot affirm whether the interaction of the antibody with the nanoparticle is purely physical or if there is a chemical bond, since the amount of antibodies used in this method are far too small to produce discernible signals in Fourier-transform infrared spectroscopy. Thus, we rely only on fluorescence data that suggest that conjugation was achieved by direct adsorption of antibodies on the AuNPs surface. Such enhancement has been rationalized in terms of competition between adsorbing species and molecular oxygen dissolved in solution, as explained before by Lima et al. [6]. We found that the replacement of ALEXA by the AuNPs in NP1 saves about 18 h (similar to A2), when compared to standard protocol and NP2 that require about 24 h, each, from the antigenic retrieval to the assembling of the slides for microscopic analysis (Fig. 7 and Table 1). The time for incubation with the primary antibody was 30 min for A2 and NP1, but overnight (18 h) for A1, NP2, and NP3 (Fig. 7a and Table 1).

Moreover, we demonstrated MIF and COX-2 primary antibody-conjugated gold nanoparticles can be arranged on the surface of M2 macrophage as a fast alternative to analyze the immune profile of inflammation-induced cancer tissues by flow cytometry. Although the standard protocol is laborious, the intensity of marking to both the primary antibodies was higher than primary antibody-conjugated gold nanoparticles. However, the primary antibody-conjugated gold nanoparticles needed less reagents and the time saving was higher as seen in the standard protocol (4 h) and in primary antibody-conjugated gold nanoparticles N1 protocol (1 h) (Fig. 7b). These results suggest that M2 macrophages can be targeted with primary antibody-conjugated gold nanoparticles either to diagnosis or therapy.

The time saving, the specificity, and the low cost provided by NP1 are especially important in cancer diagnosis, when fast and accurate results are highly required. It is important to highlight that, although the models adopted for this work were based on inflammation diseases, the inflammation markers used in this study are highly cancer-correlated and AuNPs provide multiple possibilities for application in clinical research and diagnosis.

## Conclusions

All nanoparticle protocols tested showed similar fluorescent intensities to those observed in standard IF, extending the application of AuNPs, not only in research, but also in clinical diagnostics. When diluted with ALEXA, AuNPs allow greater dilutions with acceptable fluorescence intensity. More importantly, AuNPs can be used in faster protocols (e.g., 30-min incubation protocols), completely substituting ALEXA and providing a way to develop further technologies that will improve cancer diagnose and other diseases. We believe that these findings will contribute to advance research and diagnostic procedures that utilize IF methods as well as widen the applications of AuNPs in biotechnology.

## Abbreviations

A1: ALEXA standard protocol
A2: ALEXA modified protocol
ALD: Alcoholic liver disease
ALEXA: Alexa Fluor® 488®
AuNP: Gold nanoparticles
BSA: Bovine serum albumin
COX-2: Cyclooxygenase-2
FC: Flow cytometry
FI: Fluorescence imaging
IF: Immunofluorescence
MDA: Malondialdehyde
MIF: Macrophage migration inhibitory factor
NP1: Nanoparticles protocol 1 NP2 Nanoparticles protocol 2
NP3: Nanoparticles protocol 3
SPR: Surface plasmon resonance
UC: Ulcerative colitis
